# “Caring for Those Who Care”: Staff Wellbeing in Mental Health Hospitals

**DOI:** 10.1192/bjo.2026.11353

**Published:** 2026-06-30

**Authors:** Pooja Bayyavarapu

**Affiliations:** Central and North West London NHS Trust, London, United Kingdom

## Abstract

**Aims:**

Assessing Current Well being Levels: To determine the existing levels of wellbeing among staff members working in mental health hospitals:

(1) Identifying Key Stressors: To identify the specific challenges and stressors that mental health staff encounter in their work environment.

(2) Evaluating Coping Mechanisms: To examine the coping mechanisms employed by staff to manage the emotional and psychological demands of their roles.

(3) Analysing Support Systems: To assess the effectiveness and availability of support systems, including mental health support, counselling, and debriefing opportunities for staff.

(4) Exploring Work-Life Balance: To investigate the impact of work schedules and work-life balance on staff well being.

(5) Recommendations for Improvement: To provide evidence-based recommendations for enhancing staff well being and addressing the unique challenges faced by mental health hospital staff.

These research goals aim to shed light on the well being of staff working in mental health hospitals, ultimately contributing to improved working conditions and better outcomes for both staff and the patients they look after.

**Methods:**

The research is qualitative in nature. A questionnaire consisting 12 questions, both open and close ended was curated and distributed amongst 20 participants on all wards ofThe Horton Rehabilitation Services, Central and North West London NHS Trust. The produced data was analysed using thematic analysis.

**Results:**

Theme 1: Stressors: The key stressors identified by majority of the staff were:

(1) High work load due to understaffing: Stress caused by inadequate staffing levels leading to increased workload and stress.

(2) Long working hours especially from nursing point of view has been identified.

(3) Patients displaying aggressive or violent behaviour towards staff.

(4) Inefficiencies of colleagues.

Theme 2: Self-care:

(1) Regular breaks–The importance of taking short breaks during their shifts to recharge themselves.

(2) Exercise – By engaging in physical activities as a way to maintain their mental and physical well being.

(3) Support–The value of talking to colleagues who understand the unique challenges of the job.

Theme 3: Recognition of their work:

(1) Positive patient and colleague feedback: Recognition stemming from positive feedback/testimonials from patients, their families and colleagues would encourage them to provide good quality of care.

(2) Nominations and awards: Acknowledgement through nominations for excellence or awards.

Theme 4: Training, continual development and support:

(1) Training on well being/stress management/resilience programmes. Unfortunately, none of the staff had taken training of wellbeing/stress management in the past year.

(2) Supervision: Emphasis on the importance of supervision from their line manager.

**Conclusion:**

Recommendations:

(1) Regular Well being Assessments: Implement routine assessments to gauge staff wellbeing, stress levels, and job satisfaction,if necessary anonymously. This can help identify areas of concern.

(2) Mental Health Support: Ensure access to mental health resources, such as counselling and debriefing sessions, to help staff cope with the emotional toll of their work.

(3) Stress Management Training: Provide staff with training in stress management techniques, including mindfulness and resilience-building programmes.

(4) Workload Management: Ensure manageable workloads and staffing levels to prevent burnout. Address staffing shortages to reduce stress related to high caseloads.

(5) Regular Peer Support Programmes: Establish peer support networks where staff can share experiences, seek advice, and provide emotional support to one another.

(6) Supportive Supervision: Promote regular supervision meetings where staff can discuss their work-related challenges and emotions.

(7) Staff outings and game days: Structured team-building efforts can improve team functioning, attitudes, and staff relationships, which are important for staff well being and cohesion in non-acute clinical settings.

